# A Genome-Wide Screen for Sporulation-Defective Mutants in *Schizosaccharomyces pombe*

**DOI:** 10.1534/g3.114.011049

**Published:** 2014-04-09

**Authors:** Esma Ucisik-Akkaya, Janet K. Leatherwood, Aaron M. Neiman

**Affiliations:** * Department of Biochemistry and Cell Biology, Stony Brook University, Stony Brook, New York 11794-5215; † Department of Molecular Genetics and Microbiology, Stony Brook University, Stony Brook, New York 11794-5222

**Keywords:** knockout collection, *erp2*, *erp5*, forespore membrane

## Abstract

Yeast sporulation is a highly regulated developmental program by which diploid cells generate haploid gametes, termed spores. To better define the genetic pathways regulating sporulation, a systematic screen of the set of ~3300 nonessential *Schizosaccharomyces pombe* gene deletion mutants was performed to identify genes required for spore formation. A high-throughput genetic method was used to introduce each mutant into an *h*^90^ background, and iodine staining was used to identify sporulation-defective mutants. The screen identified 34 genes whose deletion reduces sporulation, including 15 that are defective in forespore membrane morphogenesis. In *S. pombe*, the total number of sporulation-defective mutants is a significantly smaller fraction of coding genes than in *S. cerevisiae*, which reflects the different evolutionary histories and biology of the two yeasts.

Ascospore formation in yeast is a response to nutrient deficiency (Tomar *et al.* 2013). In *Schizosaccharomyces pombe*, cells exit mitosis to differentiate into spores when they encounter the lack of a nitrogen source (Tanaka and Hirata 1982; Egel 1989; Shimoda and Nakamura 2004b). First, haploid cells of opposite mating types fuse to form diploid zygotes. These diploids then immediately undergo meiosis to generate four haploid nuclei. During the course of meiosis, these nuclei become packaged into daughter cells, termed spores. Spores are created by a specialized form of cell division that occurs without cleavage of the mother cell (Shimoda 2004a). Each of the four haploid nuclei produced by meiosis are packaged into daughter cells by envelopment within newly synthesized membranes called forespore membranes (Yoo *et al.* 1973; Shimoda and Nakamura 2004b). Forespore membrane formation initiates on meiotic spindle pole bodies (SPBs) early in meiosis II and as meiosis proceeds, each forespore membrane expands to engulf the associated nucleus (Shimoda 2004a; Nakase *et al.* 2008). Closure of the forespore membrane around a nucleus completes cell division, and these cells then mature into spores by deposition of spore wall material (Yoo *et al.* 1973). All of these events occur within the cytoplasm of the original mother cell, which is referred to as the ascus.

Mutants defective in meiosis and sporulation have been identified in *S. pombe* in a number of different screens. Originally *spo* mutants were found by direct screening for sporulation defects (Bresch *et al.* 1968; Kishida and Shimoda 1986). More recently targeted mutagenesis of genes whose expression is sporulation-induced has identified additional genes involved in both processes (Gregan *et al.* 2005; Martin-Castellanos *et al.* 2005). Although these screens have defined many genes involved in sporulation, these screens were not saturating and so additional genes likely remain to be identified.

The process of sporulation is similar in *S. pombe* and in the budding yeast *Saccharomyces cerevisiae*, although there appears to be only limited conservation of the specific genes involved in the process (Shimoda 2004a). Systematic screening of the *S. cerevisiae* knockout collection has proven to be a valuable approach, identifying hundreds of genes required for sporulation (Deutschbauer *et al.* 2002; Enyenihi and Saunders 2003; Marston *et al.* 2004). Sporulation-defective mutants in *S. cerevisiae* can be divided into several broad categories: (1) genes required for aspects of cell physiology necessary to support sporulation, for example mitochondrial function or autophagy; (2) genes required for progression through meiotic prophase to the initiation of spore development; and (3) genes required for spore assembly, *per se*, for instance genes involved in growth of the prospore membrane (the *S. cerevisiae* equivalent of the forespore membrane) or for spore wall formation (Neiman 2005).

To obtain a more comprehensive list of genes required for sporulation in *S. pombe*, we undertook a genome-wide systematic screen of the *S. pombe* haploid deletion set (~3300 strains in total). In *S. pombe*, nitrogen starvation induces haploid cells of opposite mating types (*h*^+^ and *h*^−^) to mate and then undergo meiosis and spore formation. Strains that carry the *h*^90^ allele at the *mat1* locus are homothallic, meaning the cells switch mating types during mitotic growth so that both the *h*^+^ and *h*^−^ mating types are present in colonies originally derived from a single cell. Diploids generated by *h^90^* strains are therefore completely homozygous because they are a result of self-mating. This greatly simplifies the detection of meiotic and sporulation mutants because meiosis is normally induced only in diploid cells. The haploid deletion set was constructed in an *h*^+^ mating type background. Therefore, it was necessary to introduce *h^90^* into each deletion strain to enable the creation of homozygous mutant diploids. After these mutants were exposed to conditions that promote sporulation, iodine staining was used as an initial screen to determine whether spores were present (Garcia *et al.* 2006). Secondary screens included direct observation of asci by phase contrast microscopy and examination of fluorescent markers for the forespore membrane and SPBs. Our screen identified >90% of the previously known sporulation-defective mutants present in the collection, suggesting that the screen has identified the majority of non-essential genes required for spore formation. Among the novel sporulation genes are membrane trafficking proteins, signaling proteins, transcription factors, and metabolic enzymes. These results provide a wealth of information for future investigations.

## Materials and Methods

### Yeast strains and culture

Standard media and growth conditions were used unless otherwise noted (Forsburg and Rhind 2006). For synthetic medium containing G418, pombe glutamate medium (PMG) was used (Sabatinos and Forsburg 2010). Genotypes of the strains used in this study are listed in Table 1. Strain EAP20, which was used to introduce *h^90^*, as well as genes encoding tagged versions of *psy1*^+^ and *sid4*^+^ (markers for the forespore membranes and SPBs, respectively) into the knockout collection, was constructed in several steps. First, a spontaneous cycloheximide resistant mutant of strain JLP18 (EAP3) was selected by plating cells on YES plates containing 10 mg/L of cycloheximide (Sigma-Aldrich Co.). EAP11 was generated by transforming EAP3 with *Sph*I-digested pEA4, which targets integration of the *S. cerevisiae URA3* gene adjacent to the *his5*^+^ locus. *his5*^+^ is tightly linked to *mat1*, which contains the *h*^90^ allele, and the Ura^+^ phenotype can then be used to follow the *h*^90^ allele in subsequent crosses. Next, an allele of the SPB gene *sid4*^+^ fused to a gene encoding the fluorescent protein tdTomato (*sid4^+^-tdTomato*::*hphMX6*) was introduced by crossing EAP11 with strain 843 (Doyle *et al.* 2009) to generate EAP16. To introduce a marker for the forespore membrane, a strain [FY12295; (Nakase *et al.* 2008)] carrying a green fluorescent protein (GFP)-tagged allele of *psy1*^+^ was crossed to EAP16, generating EAP19. Finally, EAP19 was backcrossed to EAP16 to generate a segregant, EAP20, which carries the marked *h*^90^ locus, both fluorescent protein gene fusions, and cycloheximide resistance.

**Table 1:** Strains used in this study

Name	Genotype	Source
JLP18	*h^90^ ura4-D18 leu1-32 his3-127*	This study
EAP3	*h^90^ ura4-D18 leu1-32 his3-127 cyh^R^*	This study
EAP11	*h^90^ his5*::*URA3*::*his5^+^ ura4-D18 leu1-32 his3-127 cyh^R^*	This study
843	*h^90^ myo51^+^-GFP*::*kanMX6 sid4^+^-tdTomato*::*hphMX6 ura4-D18 leu1-32*	(Doyle *et al.* 2009)
EAP16	*h^90^ his5*::*URA3*::*his5^+^ sid4^+^-tdTomato*::*hphMX6 ura4-D18 leu1-32 his3-127 cyh^R^*	This study
FY12295	*h^90^ spo15*::*ura4^+^ ura4-D18 leu1^+^*::*GFP-psy1^+^*	(Nakase *et al.* 2008)
EAP19	*h^90^ leu1*::*GFP-psy1^+^-LEU2 sid4^+^-tdTomato*::*hphMX6 ura4-D18 leu1-32*	This study
EAP20	*h^90^ his5*::*URA3*::*his5^+^ leu1^+^*::*GFP-psy1^+^ sid4^+^-tdTomato*::*hphMX6 ura4-D18 leu1-32 cyh^R^*	This study
Bioneer deletion set	*h^+^ ade6-M210 ura4-D18 leu1-32 geneX*Δ::*kanMX4*	(Kim *et al.* 2010)
deletion mutants after outcrosses	*h^90^ his5*::*URA3*::*his5^+^ leu1^+^*::*GFP-psy1^+^ sid4^+^-tdTomato*::*hphMX6 ura4-D18 leu1-32 cyh^R^ geneX*Δ::*kanMX4*	This study

### Plasmids

pEA4, which contains the *S. pombe his5*^+^ gene in pRS306 (Sikorski and Hieter 1989), was constructed by polymerase chain reaction (PCR) amplification of a 1.3-kb fragment including *his5*^+^ and its 5′ and 3′ regions from genomic DNA using EAO11 (5′-GTTCTTGGTACCGAGCGTGCTCAGTTTTCTATG-3′) and HJO274 (5′-GTTGTTGAA TTCTTACAACACTCCCTTCGTGCTTGGG-3′) oligonucleotides. The PCR product was engineered to contain *Kpn*I and *Eco*RI sites at its 5′ and 3′ ends, respectively, and was cloned into similarly digested pRS306.

pEA18, which expresses *wsc1*^+^-*mTagBFP* under control of the *spo13* promoter, was constructed in three steps. First, a yeast codon-optimized form of mTagBFP without a stop codon was PCR amplified from pRS426 *Spo20^51–91^-mTagBFP* (Lin *et al.* 2013) using EAO44 (5′-GTTCTTCATATGGTTCTTGTTCCATGGATGTCTGAGGAGTTGATAAAGG-3′) and EAO46 (5′-GTTCTTGGATCCCTTGTTCTTGCGGCCGCGTTCAACTTGTGACCCAACTTTG-3′) oligos and cloned as a *Nde*I/*Bam*HI fragment into similarly digested pREP42x (Forsburg 1993) creating pEA13. Second, overlap PCR was used to construct a P*spo13-wsc1^+^* fusion. A ~500-bp fragment of the *spo13* promoter region and the *wsc1^+^* open reading frame lacking the stop codon were amplified using the oligonucleotide pairs EAO47 (5′-GTTCTTCTGCAGGGCACTCTGTAATTGTAAG-3′) and EAO48 (5′-GAGGAATTTAAAAAGACCATAGATCTTGTTTCAATTTTTTTTCCTTTCC-3′), and EAO49 (5′-GGAAAGGAAAAAAAATTGAAACAAGATCTATGGTCTTTTTAAATTCCTC-3′) and EAO50 (5′-GTTCTTCCATGGGTTCAAATTTGTGACACGC), respectively. These PCR products were mixed and used as template in a reaction with EAO47 and EAO50 to yield a ~1.6-kb *spo13pr-wsc1^+^* fusion fragment. This product was digested with *Pst*I and *Nco*I and cloned into similarly digested pEA13 to replace the *nmt1* promoter of pREP42x in front of mTagBFP creating pEA17. Finally, pEA18 was created by amplifying mTagBFP with its stop codon from pRS426 *Spo20^51–91^-mTagBFP* using EAO44 and EAO45 (5′-GTTCTTGGATCCCTTGTTCTTGCGGCCGCTTAGTTCAACTTGTGACCCAACTTTG-3′), digested with *Nco*I and *Not*I, and cloned into similarly digested pEA17.

### Genetic screen

**Figure 1:**
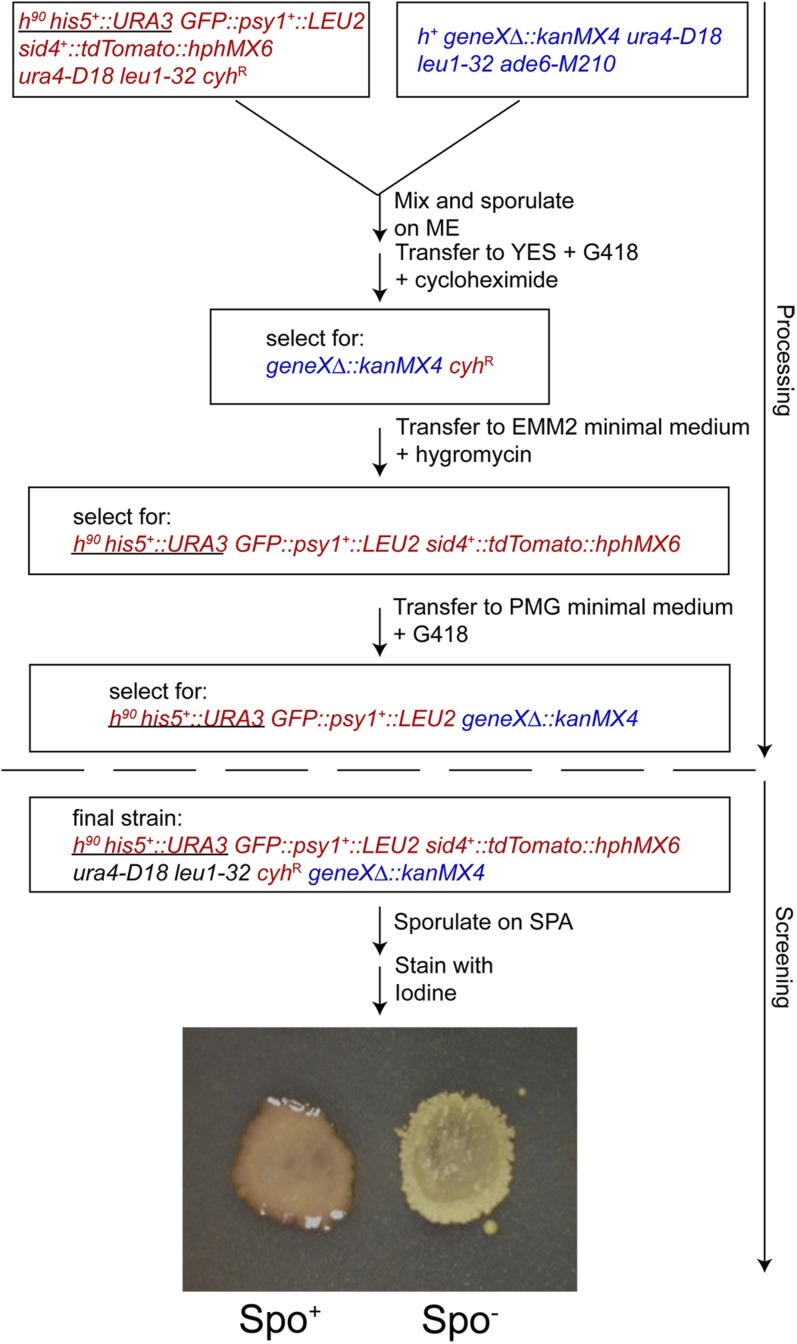
Outline of generation of homothallic mutant strains and the screen for sporulation defective mutants. Genotypes of cells at each stage are boxed. Blue indicates genes from the deletion set strains, and red indicates genes derived from EAP20. These two strains were first mixed in liquid and then spotted onto ME plates to allow mating and sporulation. Cells were then replica plated to plates containing G418 and cycloheximide to select for recombinant progeny containing both the *geneX*Δ::*kanMX4* and the *cyh*^R^ alleles. These haploids were transferred to minimal medium containing hygromycin to select for those segregants that also carry the *h^90^* mating type (linked to *URA3*, indicated by the underline) and harbor markers for the forespore membrane (*GFP-psy1^+^*) and the SPB (*sid4^+^-tdTomato*::*hphMX6*). A final transfer to minimal medium containing G418 ensures that the deletion alleles are still present. Meiosis and sporulation were induced by replica plating the patches to SPA medium and spore formation was assayed by exposure of the cells to iodine vapor, which causes spores to exhibit a red colony color. An example of sporulation proficient and defective patches is shown.

The haploid *S. pombe* deletion mutant library was purchased from Bioneer (South Korea). The knockouts are in an *h*^+^ strain background (*h*^+^ *ade6-M210 ura4-D18 leu1-32*). To examine sporulation, each mutant was crossed to strain EAP20 and a modified form of the synthetic gene array method was used to introduce the knockout alleles into an *h*^90^ background (Tong *et al.* 2001; Baryshnikova *et al.* 2010). The steps in this process are outlined in Figure 1. First, strains containing individual *geneX*Δ::*kanMX4* deletions were grown in liquid YES medium in microtiter dishes. To each well was then added 1/10th volume of a saturated culture of EAP20 grown in YES and the mixed cultures were pinned onto ME plates, allowing the cells to grow, mate, and sporulate (Forsburg and Rhind 2006). Use of ME at this step produced higher efficiency sporulation than other media (data not shown). The patches were then replica plated to YES plates supplemented with 200 mg/L Geneticin (G418; USBiological Life Sciences, Salem, MA) and 10 mg/L cycloheximide. Combined, these drugs select for recombinant haploids from the cross. Geneticin selects for the knockout marker. Cycloheximide resistance is a recessive trait, and cycloheximide therefore selects against both the original knockout strain and any diploid cells created by mating of EAP20 with the deletion strain. Inclusion of this step is essential to prevent a background of diploids heterozygous for the knockout allele from contaminating the patches (Baryshnikova *et al.* 2010). After 3 d incubation at 31°, patches were replica plated to EMM2 plates with 200 mg/L Hygromycin B (Calbiochem, Merck KGaA, Darmstadt, Germany). This medium selects for both uracil and leucine prototrophy, which are linked to *h*^90^ and the forespore membrane marker GFP-*psy1^+^*, respectively, and for the SPB marker *sid4^+^-tdTomato*::*hphMX6*. In addition it also selects against the *ade6-M210* allele found in the deletion set. It is important that the resulting strain be *ade6*^+^ as the red pigment created by the *ade6-M210* mutant complicates both the subsequent iodine staining and fluorescence analyses. We found that removal of the G418 selection at this step allowed the growth of cells lacking the knockout allele and so an additional replica plating to PMG plates with 200 mg/L G418 was performed. G418 selection is more efficient on PMG than EMM2 (Benko and Zhao 2011). The resulting patches consist of *h*^90^ haploid deletion mutants harboring *sid4^+^-tdTomato*::*hphMX6* and GFP-*psy1^+^*. These patches were then replica plated to SPA plates to induce sporulation, incubated at 25° for 3 d, and then inverted over a Petri dish of iodine crystals for 2-3 min (Sabatinos and Forsburg 2010). Staining of mature spore wall with iodine vapor produces a dark brown color (Meade and Gutz 1975). Patches displaying absent or reduced staining with iodine were scored as sporulation-defective candidates.

Candidates were picked from the PMG plates to a fresh PMG plate with 200 mg/L G418, replica plated to SPA, and then retested for iodine staining. Cells from patches that failed to exhibit good iodine staining after the retest were then directly analyzed by light microscopy for the presence of visible spores and by fluorescence microscopy of the Sid4-tdTomato and GFP-Psy1 markers to evaluate progression through meiosis and forespore membrane formation.

### Barcode sequencing

In construction of the knockout collection, each knockout incorporated “uptag” and “downtag” sequences that provide a unique barcode for each knockout (Kim *et al.* 2010). To confirm the identity of the mutants identified in our screen, we amplified the uptag region for each one. This PCR product was then sequenced using EAO62 (5′-GGGCGACAGTCACATCATGC3′-) and the results were compared with the list of uptag sequences given by Kim *et al.* (2010). In addition, the *meu14* and *mfr1* deletions were also analyzed by PCR-amplification of the loci with flanking primers to distinguish the knockout and wild-type alleles.

### Microscopy

Images were collected on a Zeiss Observer Z.1 microscope and processed using Zeiss Axiovision or Zen software.

### Acetone resistance assays

Spore wall function was tested using an acetone resistance assay modified from (Smith 2009). Wild-type spores are resistant to acetone, whereas unsporulated cells or cells with defective spore walls are killed. The wild-type and the knockout strains were first incubated on PMG plates with 200 mg/L G418 at 31° for 2 d. Cells were then replica plated to SPA plates and incubated at 25° for 3 d to allow for mating and sporulation, and then replica-plated onto YES plates. An acetone-soaked filter paper (Whatman #3, 1003-090) was placed on a glass Petri dish and inverted above the YES plate to expose the patches to acetone vapor for 15 min. These were then incubated at 31° for 3 d before being photographed.

## Results and Discussion

### Isolation of sporulation-defective mutants

Using a series of selective steps diagrammed in Figure 1, we constructed *h^90^* homothallic derivatives of each deletion strain in the Bioneer *S. pombe* haploid deletion collection, at the same time introducing fluorescent markers for the SPBs and the forespore membrane. Because *h^90^* strains are able to undergo mating type switching, *h^90^* cells can be induced to self-mate and create homozygous diploids that then proceed through meiosis and sporulation. The ability of the strains in the deletion set to form spores was then assayed by exposure to iodine, which produces a dark brown stain in patches containing spores.

Eighty-five candidates passed the initial screen as well as a retest. In addition to sporulation-defective mutants, the assay of decreased iodine staining might also identify knockouts that cause *h*^−^-specific mating defects, that is, mutants that are unable to mate with *h^+^* haploids. The deletion strain background is *h^+^* and these cells are therefore able to mate with *h*^-^ cells present in the *h^90^* background in the initial cross. However, these cells will be unable to self-mate once in the *h^90^* background and so will not produce spores. Similarly, as the *URA3* marker is integrated approximately 10 cM from the *mat1* locus containing the *h^90^*allele (Egel 2004), recombination between *URA3* and the *mat* locus can produce *URA3 h^+^* haploids that would slip through the selection procedure and these would also fail to sporulate. To test for such false positives, the 85 strains were assayed for their mating types by replica-plating to *h^+^* and *h^−^* tester strains followed by iodine staining to examine whether diploids formed that could sporulate. Strains that are *h^90^* are expected to mate to both *h^−^* and *h^+^* cells. Three of the candidates mated only to the *h^+^* tester strains, and thirty-three mutants mated only to the *h^−^* tester, demonstrating that a mating defect is indirectly responsible for the absence of spores. Of the strains that mated only to the *h^−^* tester, two were deletions in *mam1* (M-factor transporter) and *mam2* (P-factor receptor), both of which are known to produce an *h*^−^ specific sterility (Kitamura and Shimoda 1991; Christensen *et al.* 1997). The remaining mutants we suspect were simply *h*^+^ strains that leaked through the selection process. The strains with mating defects were not analyzed further.

The remaining candidates were sporulated and examined by phase contrast microscopy to determine the frequency of spore formation in the culture. Those strains in which no spores were detected were also examined by fluorescence microscopy of the Sid4-tdTomato and GFP-Psy1 markers to look at progression through meiosis and forespore membrane formation, respectively. Based on these microscopy assays the mutants can be divided into four classes: (1) reduced frequency of zygotes, suggesting that the sporulation defect is secondary to a mating defect; (2) near wild-type frequency of zygotes and spores, suggesting a defect in formation of the iodine-reactive layer of the spore wall; (3) no spores and no forespore membrane formation; and (4) no spores with abnormal forespore membrane formation (Table 2).

**Table 2:** Genes identified in the sporulation-defective screen

Gene	Gene ID	Comments*^a^*
Class 1. Genes required for zygote formation (n = 7)		
* atg12*^+^	SPAC1783.06c	Autophagy-associated ubiquitin-like modifier
* cyp9*^+^	SPCC553.04	Predicted cyclophilin family peptidyl-prolyl *cis*-trans isomerase
* mmd1*^+^	SPAC30C2.02	Predicted deoxyhypusine hydroxylase
* prm1*^+^	SPAP7G5.03	Integral membrane protein important for cell−cell fusion
	SPBC1711.12	Predicted oxidized protein hydrolase
	SPBC18E5.08	Predicted *N*-acetyltransferase
	SPBC146.02	Sequence orphan
Class 2. Genes required for spores to be iodine-reactive (n = 7)		
* fsc1*^+^	SPAC22H12.05c	Fasciclin domain protein
* lcf2*^+^	SPBP4H10.^11^C	Long-chain-fatty-acid-CoA ligase
* mam3*^+^	SPAP11E10.02c	Cell agglutination protein
* mcl1*^+^	SPAPB1E7.02c	DNA polymerase alpha accessory factor
* php3*^+^	SPAC23C11.08	CCAAT-binding factor complex subunit
* php5*^+^*^b^*	SPBC3B8.02	CCAAT-binding factor complex subunit
* rik1*^+^	SPCC11E10.08	Silencing protein
Class 3. Genes required for entry into meiosis or for the initiation of forespore membrane assembly (n = 5)		
* mei2*^+^	SPAC27D7.03c	RNA-binding protein required for meiosis
* mei3*^+^	SPBC119.04	Required for the initiation of meiosis
* mei4*^+^	SPBC32H8.11	Transcription factor regulating meiotic gene expression
* mug79*^+^ *(spo7*^+^*)*	SPAC6G9.04	Meiotic spindle pole body component
* spo15*	SPAC1F3.06c	Meiotic spindle pole body component
Class 4. Genes that are essential for the proper formation and the maturation of the forespore membrane (n = 15)		
* csn1*^+^	SPBC215.03c	COP9/signalosome complex subunit
* csn2*^+^	SPAPB17E12.04c	COP9/signalosome complex subunit
* cdt2*^+^	SPAC17H9.19c	COP9/signalosome associated factor
* erp2*^+^	SPAC17A5.08	ER exit receptor for secretory cargo
* erp5*^+^	SPBC16E9.09c	ER exit receptor for secretory cargo
* mes1*^+^	SPAC5D6.08c	Meiotic APC/C regulator
* spe2*^+^	SPBP4H10.05c	S-adenosylmethionine decarboxylase proenzyme
* (spe3*^+^*)*	SPBC12C2.07c	Predicted spermidine synthase
* spn2*^+^	SPAC821.06	Septin
* spo3*^+^	SPAC607.10	Required for spore formation
* spo4*^+^	SPBC21C3.18	Kinase required for spore formation
* spo5*^+^	SPBC29A10.02	Meiotic RNA-binding protein
* tpp1*^+^	SPAC19G12.15c	Trehalose-6-phosphate phosphatase
	SPAC6C3.06c	Predicted P-type phospholipid flippase
	SPCC1739.04c	Sequence orphan

ER, endoplasmic reticulum; APC/C, Anaphase Promoting Complex/Cyclosome.

a Descriptions are based on PomBase (Wood *et al.* 2012) (www.pombase.org).

b Knockout not confirmed by barcode sequencing.

To confirm the identity of the deleted gene in the knockout strains, we used PCR to amplify the unique uptag region for many of the deletions (Kim *et al.* 2010). These PCR products were then sequenced and compared with the published lists to confirm the identity of the knockouts. For 48 knockouts for which we obtained sequences, 32 matched the published barcodes. The knockouts that did not produce the expected barcode sequence are listed in Table 3. In three cases, the barcode sequence found corresponded to that of known sporulation-defective mutants, suggesting that the identification by the barcode sequence, rather than position in the collection, is correct. In all cases of misidentification, the expected knockout and the actual one are found in different plates within the collection. These errors are, therefore, unlikely to have been caused by cross-contamination during our handling of the collection as different plates were processed at different times. Although this is a small sample, the surprisingly high error rate (33%) highlights the need for confirmation of knockout identity when using this collection.

**Table 3:** Gene deletions that do not have the correct barcode

Gene	Gene ID	Barcode Information
*atg15*Δ	SPAC23C4.16c	Matches with *spo5*
*atp10*Δ	SPAC4G8.11c	No match
*atp14*Δ	SPBC29A3.10c	No match
*ctf1*Δ	SPBC3B9.11c	No match
*lsk1*Δ	SPAC2F3.15	Matches with *mei4*
*mei4*Δ	SPBC32H8.11	No match
*mfr1*Δ	SPBC1198.12	Matches with SPAC17H9.14c *mfr1* knockout is not present as determined by PCR with flanking primers,
*scd1*Δ	SPAC16E8.09	Matches with *mei4*
*spo5*Δ	SPBC29A10.02	No match
*spo6*Δ	SPBC1778.04	No match
	SPBC15C4.06c	No match
*apq12*Δ	SPBC428.04	Matches with *cyp9*
	SPBC21H7.06c	Matches with *cyp9*
	SPAC139.01c	Matches with *nrd1*
	SPBC23G7.06c	Matches with *nrd1*
	SPBC1711.08	Matches with *nrd1*

To test the effectiveness of our screen, we culled from the literature a list of previously identified mutants that block spore formation. Several of the original *spo* mutants proved to be hypomorphic alleles of essential genes (Nakase *et al.* 2001; Nakamura-Kubo *et al.* 2003) and so are not present in our deletion set; however we identified 13 known sporulation-defective mutants listed as present in the collection (Table 4). Amplification and barcode sequencing confirmed the presence of nine of these at the correct location in the collection and another two mutants were identified at different locations. Of these 11 mutants, 10 were identified in the screen. This yield suggests that the screen has identified ~90% of the sporulation-defective mutants present in the collection.

**Table 4:** Known sporulation-defective genes listed in the *S. pombe* haploid deletion set

Gene	Comment	Phenotype in Our Study
*spo3*^+^	Confirmed by barcode sequence	Sporulation defect
*spo4*^+^	Confirmed by barcode sequence	Sporulation defect
*spo5*^+^	Knockout found at different position in the collection*^a^*	Sporulation defect
*spo6*^+^	Not present*^a^*	n.d.*^b^*
*mug79*^+^*/spo7*^+^	Confirmed by barcode sequence	Sporulation defect
*spo15*^+^	Confirmed by barcode sequence	Sporulation defect
*mei2*^+^	Confirmed by barcode sequence	Sporulation defect
*mei3*^+^	Confirmed by barcode sequence	Sporulation defect
*mei4*^+^	Knockout found at different position in the collection*^a^*	Sporulation defect
*mes1*^+^	Confirmed by barcode sequence	Sporulation defect
*mfr1*^+^	Not present*^a^*	n.d.
*meu14*^+^	Knockout is present as determined both by PCR with flanking primers and by barcode sequence	Normal sporulation
*cdt2*^+^	Confirmed by barcode sequence	Sporulation defect

n.d., not determined; PCR, polymerase chain reaction.

a See Table 3.

### Classes of genes required for positive iodine staining phenotype

#### Genes required for zygote formation:

For mutants that formed some level of visible spores, the frequency of zygote formation and of spore formation were examined by light microscopy (Table 5). Mutants that display bilateral mating defects, that is, are able to mate with the *h^+^* and *h^−^* tester strains but are unable to self-mate to produce zygotes would pass the mating tests described above and show reduced sporulation. For seven mutants, zygote formation was reduced greater than threefold from that seen in a wild-type *h*^90^ strain, indicative of a bilateral mating defect. Thus, the primary defect in these mutants is likely to be in the mating process or response to nitrogen starvation rather than in spore formation, *per se*. The two genes in this class with the strongest phenotype were *prm1*^+^ and *cyp9*^+^. Consistent with our interpretation, *prm1*^+^ encodes an integral membrane protein recently shown to be necessary for conjugation (Sun *et al.* 2013; Curto *et al.* 2014). These results reveal a previously unknown role for *cyp9*^+^ in the mating reaction.

**Table 5:** Mating and sporulation efficiency of different mutants

Gene	Gene ID	Class*^a^*	% of Zygotes*^b^* (SD)	% of Sporulation^c^ (SD)
WT			67.0 (4.0)	76.7 (5.5)
*cyp9*Δ	SPCC553.04	1	<0.5	n.d.
*prm1*Δ	SPAP7G5.03	1	<0.5	n.d.
	SPBC1711.12	1	9.0 (2.2)	21.3 (1.2)
	SPBC18E5.08	1	9.8 (2.4)	87.7 (2.5)
	SPBC146.02	1	14.8 (5.0)	3.3 (0.6)
*atg12*Δ	SPAC1783.06c	1	19.0 (8.6)	47.7 (6.8)
*mmd1*Δ	SPAC30C2.02	1	21.5 (11.2)	71.0 (8.9)
*fsc1*Δ	SPAC22H12.05c	2	27.3 (12.6)	52.0 (4.4)
*mcl1*Δ	SPAPB1E7.02c	2	38.5 (8.3)	56.3 (12.5)
*php3*Δ	SPAC23C11.08	2	38.5 (6.6)	43.0 (6.6)
*mam3*Δ	SPAP11E10.02c	2	39.5 (4.8)	76.3 (6.7)
*lcf2*Δ	SPBP4H10.^11^C	2	43.8 (11.5)	58.5 (6.1)
*rik1*Δ	SPCC11E10.08	2	57.8 (7.1)	85.3 (3.9)

SD, standard deviation; n.d., not determined.

a Class 1 = Genes required for zygote formation; Class 2 = Genes required for spores to be iodine-reactive.

b The average of at least three experiments. At least 100 cells were counted in each experiment.

c The average of at least three experiments. At least 100 asci were counted in each experiment.

#### Genes required for spores to be iodine-reactive:

Mutants in seven additional genes formed zygotes at near normal frequency and displayed at most modestly reduced spore formation relative to wild type. Because strains in this class form significant numbers of spores, their loss of staining may reflect defects in generation of the iodine reactive alpha-glucan component of the spore wall (Garcia *et al.* 2006). It is noteworthy that a number of mutants known to disrupt assembly of the beta-glucan or chitosan layers of the spore wall were present in the collection but were not found in our screen, probably because those mutants that effect beta-glucan or chitosan do not alter iodine staining (Liu *et al.* 2000).

Two of the genes in this class, *php3*^+^ and *php5*^+^, encode subunits of the CCAAT-binding transcription complex (McNabb *et al.* 1997; Mercier *et al.* 2006). Although previous reports have implicated this complex in induction of transcription during nitrogen starvation and in the activity of meiotic recombination hotspots, no requirement for these genes in spore formation has been reported (Nakashima *et al.* 2002; Steiner *et al.* 2011). This work suggests that transcriptional induction by this complex of as yet unidentified genes is important for proper spore formation.

#### Genes required for entry into meiosis or for the initiation of forespore membrane assembly:

The five genes identified in this class were previously known. Three of the genes are required for entry into meiosis. *mei2*^+^ encodes an RNA-binding protein that is required for premeiotic DNA synthesis as well as progression into meiosis I (Watanabe *et al.* 1988; Watanabe and Yamamoto 1994). *mei3*^+^ is essential for the initiation of meiosis since it encodes a protein that binds and inhibits the meiosis-inhibitory protein kinase Pat1 during sporulation(McLeod and Beach 1988). The transcription factor that is encoded by *mei4*^+^ is a regulator necessary for the expression of many sporulation-induced genes (Horie *et al.* 1998). The remaining two genes in this class, *mug79*^+^/*spo7*^+^ and *spo15*^+^, both encode components of the meiotic SPB necessary for the SPB to catalyze the coalescence of secretory vesicles into a forespore membrane (Ikemoto *et al.* 2000; Nakamura-Kubo *et al.* 2011).

#### Genes that are essential for the proper formation and the maturation of the forespore membrane:

Mutants in Class 4 genes progress through meiosis and initiate forespore membrane growth, but the membranes display morphological defects and no spores are visible by light microscopy. There were 15 genes identified in this category, of which five (*spo3*^+^, *spo4*^+^, *spo5*^+^, *mes1*^+^, *spn2*^+^) were previously shown to be required for sporulation (Nakamura *et al.* 2001, 2002; Izawa *et al.* 2005; Kasama *et al.* 2006; Onishi *et al.* 2010). Among the 10 genes in this class not previously associated with sporulation defects, two encode subunits of the COP9 signalosome (*csn1*^+^ and *csn2*^+^) and one encodes a reported interacting partner of the signalosome (*cdt2*^+^) (Mundt *et al.* 1999; Liu *et al.* 2005). Several other COP9 subunits are present in the collection but were not found to be iodine-negative in our screen. Thus, the Csn1 and Csn2 subunits of the signalosome may be specifically required for sporulation. A similar difference in function between Csn1/Csn2 and other COP9 subunits in sensitivity to DNA damage has been noted previously (Mundt *et al.* 2002). Also in this class are *spe2*^+^ and SPBC12C2.07c (*spe3*^+^), genes that encode enzymes involved in consecutive steps in spermidine synthesis (Tabor and Tabor 1985; Chattopadhyay *et al.* 2002). This pathway has also been shown to be required for sporulation in *S. cerevisiae* (Cohn *et al.* 1978), suggesting a conserved requirement for spermidine for spore formation in fungi.

Three of the mutants in this class have predicted functions within the secretory pathway. SPAC6C3.06c encodes a predicted phospholipid flippase orthologous to the *NEO1* gene of *S. cerevisiae*. Neo1 is localized to the endosome and to the Golgi and has been implicated in membrane trafficking (Hua and Graham 2003; Wicky *et al.* 2004). The *erp2*^+^ and *erp5*^+^ genes encode two *S. pombe* members of the p24 protein family. The p24 proteins are a highly conserved family of integral membrane proteins that act as cargo receptors and shuttle between the endoplasmic reticulum (ER) and the Golgi (Strating and Martens 2009). In particular, they play an important role in cargo selection and packaging into COPII vesicles at ER exit sites (Strating and Martens 2009). Consistent with the similar phenotypes of both *erp2* and *erp5* deletions, studies in *S. cerevisiae* suggest that the four family members function in a single complex (Hirata *et al.* 2013). Knockouts of the other family members in *S. pombe*, *emp24*^+^ (SPCC24B10.17.1) and *erv25*^+^ (SPAC23H4.03c.1), were not present in the collection, though we expect mutants in these genes would display a similar sporulation defect. We predict that the p24 family is necessary for the exit of some protein(s) from the ER so that the cargo protein(s) can be transported through the secretory pathway to the forespore membrane and contribute to proper membrane growth.

### *erp2* and *erp5* mutants do not cause a general block to ER exit

**Figure 2:**
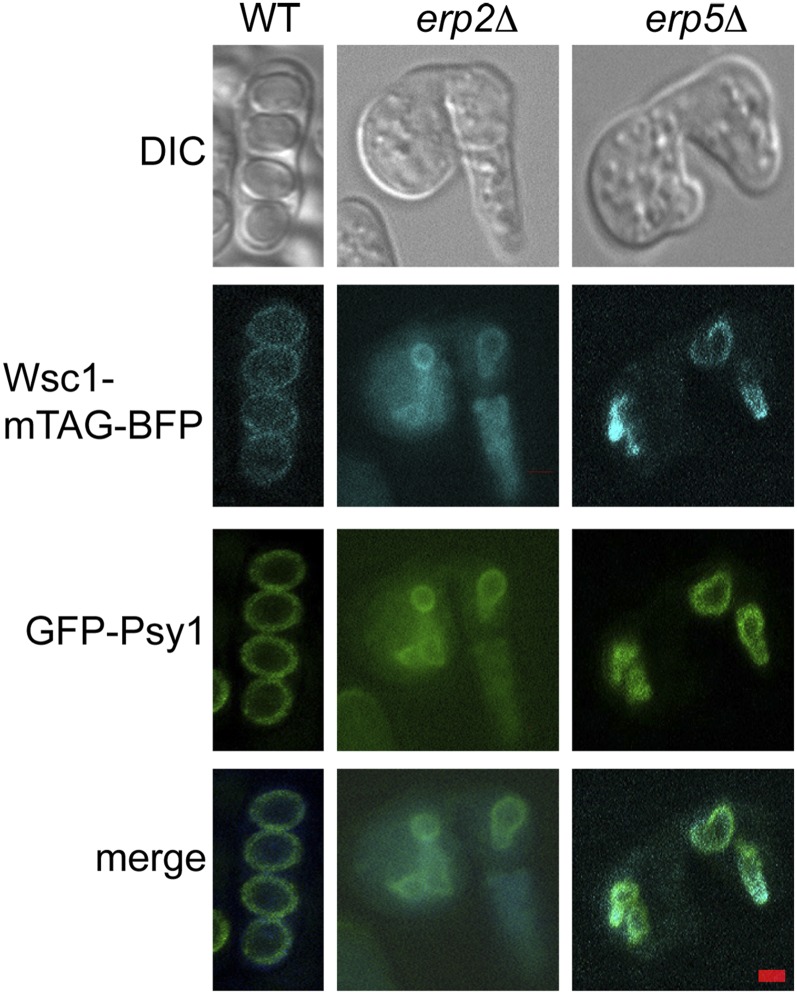
Wsc1-mTagBFP localization in *erp2* and *erp5* mutants. Wild-type, *erp2*Δ, or *erp5*Δ cells expressing *P_spo13_-wsc1^+^-mTagBFP* and *GFP-psy1^+^* were imaged after 24-hr incubation on SPA plates. Scale bar = 2 microns.

In *S. cerevisiae*, a different class of ER cargo receptor, encoded by the *ERV14* and *ERV15* genes, is required for proper formation of the prospore membrane (the budding yeast equivalent of the forespore membrane) during sporulation (Powers and Barlowe 1998; Nakanishi *et al.* 2007). Although these genes are not essential for vegetative growth, their deletion creates a general block to ER exit of integral membrane proteins during sporulation (Nakanishi *et al.* 2007). Because *ERV14* deletion mutants have small, abnormal forespore membranes similar to *erp2Δ* and *erp5Δ* mutants, we hypothesized that, parallel to the *S. cerevisiae* ER cargo receptors, *erp2*^+^ and *erp5*^+^ might become essential for ER exit of integral membrane proteins in *S. pombe* sporulation. The GFP-Psy1 reporter is localized to the forespore membrane in *erp2*Δ and *erp5*Δ cells, however this does not provide a strong test of a role for *erp2*^+^ and *erp5*^+^ in ER exit as Psy1 is relocalized from the plasma membrane to the forespore membrane via the endosome (Kashiwazaki *et al.* 2011). Therefore, to test a possible general role for *erp2*^+^ and *erp5*^+^ in ER exit, the strains were transformed with a plasmid carrying an integral plasma membrane protein, *wsc1*^+^, fused with mTagBFP and placed under control of the sporulation-specific protein *spo13* promoter (Nakase *et al.* 2008). When expressed in a wild-type strain, Wsc1-mTagBFP localized to the forespore membrane (Figure 2). In *erp2*Δ and *erp5*Δ mutants Wsc1-mTagBFP colocalized with GFP-Psy1 in the abnormal forespore membranes and no additional BFP fluorescence from the ER was seen, indicating that transport of Wsc1-mTagBFP is unaffected in the mutants (Figure 2). If loss of *erp2* or *erp5* cause forespore membrane defects indirectly by limiting the exit of some cargo from the ER, this is likely an effect on some specific cargo protein(s) and not due to a more general block in transport.

### The *lcf2*^+^ and *mcl1*^+^ gene products may contribute to spore wall function

**Figure 3:**
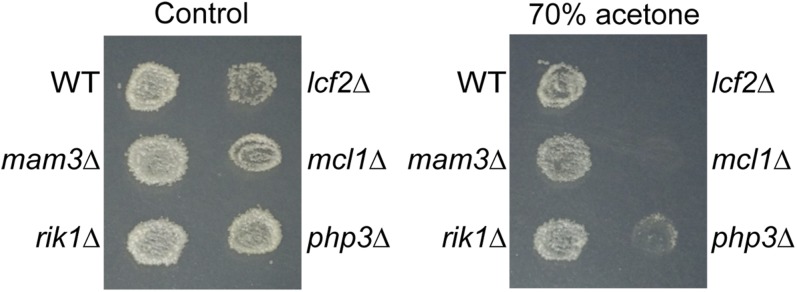
Acetone resistance assay. Indicated mutants were sporulated on SPA plates and then replicated to YES plates. Left: growth without exposure to acetone. Right: strains were exposed to 70% acetone for 15 min before incubation at 31° for 3 days.

The spore wall provides the cell with resistance to environmental stresses such as acetone vapor (Egel 1977). To examine spore wall function we tested mutants in Class 2 for resistance to acetone (Smith 2009). Two of the mutants, *lcf2*Δ and *mcl1*Δ, showed strong sensitivity to acetone exposure (Figure 3). This stress-sensitivity is striking as these mutants show near-normal levels of sporulation. This result suggests a structural defect in the spore walls of these mutants, presumably in the alpha-glucan component of the spore wall. The *mcl1*^+^ gene encodes a polymerase alpha accessory protein, so its effect on the spore wall is likely indirect (Williams and McIntosh 2005). *lcf2*^+^ encodes a predicted fatty-acyl CoA ligase, which could influence the composition of cellular membranes (Fujita *et al.* 2007). The stress sensitivity and iodine staining defects in these cells may reflect an influence of *lcf2*^+^ on the activity or delivery of the integral membrane Mok14 alpha-glucan synthase responsible for synthesis of the iodine-reactive polymer (Garcia *et al.* 2006).

### Sporulation genes in budding and fission yeast

In this screen we have produced the first survey of the nonessential knockout collection of *S. pombe* for sporulation defective mutants. One of the most striking results is the relatively small number of mutants that displayed a sporulation defect. In all, only ~1% of the *S. pombe* collection showed loss or reduction of spores. This low number is not due to poor recovery in our screen, as we identified known mutants with ~90% efficiency. By contrast, comparable screens of the knockout collection in *S. cerevisiae* found that more than 10% of the knockouts produced a sporulation defect (Enyenihi and Saunders 2003; Marston *et al.* 2004). In part, the reasons for this difference reflect the different biology of these two yeasts. Whole categories of genes essential for sporulation in *S. cerevisiae* are not found in our screen. For example, *S. cerevisiae* is a petite-positive yeast that can grow in glucose medium without functional mitochondria (Kominsky and Thorsness 2000). However, sporulation is an obligatorily aerobic process. Therefore, any mutations that impair respiration are viable but sporulation defective. This accounts for more than a quarter of the sporulation-defective mutants in *S. cerevisiae* (Neiman 2005). In contrast, *S. pombe* cannot grow mitotically without mitochondrial function, and so most of the orthologous genes should be essential in fission yeast (and therefore absent from the haploid deletion set). Another significant fraction of sporulation-defective genes in budding yeast are involved in autophagy, either directly or through effects on vacuolar function (Neiman 2005). Though autophagy mutants were present in the *S. pombe* deletion set, only one, *atg12*, was iodine-negative and it displayed reduced zygote formation, indicating a role in mating, not sporulation (Table 2). This result is consistent with previous studies showing that many autophagy mutants display reduced mating, but if mutant cells succeed in mating they are capable of sporulation (Kohda *et al.* 2007; Sun *et al.* 2013). Finally, in *S. cerevisiae*, mutations that cause defects in meiotic recombination can lead to activation of a checkpoint that arrests cells in meiotic prophase thereby preventing them from producing spores (Roeder and Bailis 2000). Although the orthologous genes and meiotic recombination checkpoint are present in *S. pombe*, failures in meiotic recombination in fission yeast lead only to a delay in meiotic progression (Shimada *et al.* 2002; Perez-Hidalgo *et al.* 2003). Thus, these mutants eventually do form spores and so were not found in our screen.

Even when we accounted for the absence of these three categories of mutants, there appear to be fewer nonessential genes required for spore assembly in *S. pombe* than in *S. cerevisiae*. This finding probably reflects the different evolutionary histories of the yeasts. The whole-genome duplication that occurred during the evolution of *Saccharomyces* allowed for the emergence of distinct sporulation- and vegetative-specific isozymes (Wolfe and Shields 1997). For example, the t-SNAREs *psy1*^+^ and *sec9*^+^ are both essential genes in *S. pombe* that are also essential for forespore membrane growth (Nakamura *et al.* 2005; Maeda *et al.* 2009). In *S. cerevisiae*, gene duplication has produced two versions of each gene, *SSO1*/*SSO2* for *psy1*^+^ and *SEC9*/*SPO20* for *sec9*^+^. In each case, one paralog is specifically required for sporulation (*SSO1* and *SPO20*) (Neiman 1998; Jantti *et al.* 2002). In the absence of such extensive gene duplication, there has been less opportunity for sporulation-specific functions to evolve in *S. pombe* and as a result, we expect that more essential genes play “double duty” in both vegetative growth and sporulation.

In summary, we report the results of a systematic screen through the *S. pombe* haploid deletion set for mutants displaying spore formation defects. The genes identified provide multiple new avenues for investigation into spore differentiation. These include a role for the COP9 signalosome in forespore membrane formation, sporulation-specific requirements for the p24 family of ER export cargo receptors, and the possible function of fatty acid metabolism in regulation of spore wall assembly.
